# Antifungal Activity of the Essential Oil of *Echinops kebericho* Mesfin: An *In Vitro* Study

**DOI:** 10.1155/2020/3101324

**Published:** 2020-11-12

**Authors:** Tamirat Bekele Beressa, Serawit Deyno, Paul E. Alele

**Affiliations:** ^1^Department of Pharmacology and Therapeutics, Faculty of Medicine, Mbarara University of Science and Technology, P.O. Box 1410, Mbarara, Uganda; ^2^Pharm-biotechnology and Traditional Medicine Center, Mbarara University of Science and Technology, Mbarara, Uganda; ^3^Department of Pharmacy, College of Medicine and Health Sciences, Ambo University, Ambo, Ethiopia; ^4^Department of Pharmacology, School of Pharmacy, College of Medicine and Health Sciences, Hawassa University, Hawassa, Ethiopia

## Abstract

**Background:**

*Echinops kebericho* is an endemic medicinal plant in Ethiopia widely used in the treatment of infectious and noninfectious diseases. Essential oils are known for their antibacterial, antifungal, antiviral, insecticidal, and antioxidant properties. This study evaluated the antifungal activity of essential oil from *E. kebericho* against four common pathogenic fungi and two standard strains.

**Methods:**

The essential oil was obtained by hydrodistillation. The antifungal screening was done by agar well diffusion method. Minimal inhibitory concentrations (MICs) were determined by broth microdilution. Minimal fungicidal concentrations (MFCs) were determined by subculturing fungal strains with no visible growth onto a Sabouraud dextrose agar (SDA) plate.

**Results:**

*Candida albicans and Cryptococcus neoformans* were highly sensitive while *Aspergillus flavus* did not show sensitivity up to 1 mg/ml of essential oil; MICs ranged from 0.083 mg/ml to 0.208 mg/ml. Concentration and fungal species showed significant dose-dependent associations (*p* < 0.0001) with antifungal activity. The MICs of essential oil were comparable to those of the standard drug (fluconazole) against *C. glabrata* and *C. krusei.* The lowest MFC of the essential oil was observed against *Candida parapsilosis* (0.145 mg/ml) while the highest MFC was against *Candida krusei* (0.667 mg/ml).

**Conclusion:**

*Echinops kebericho* essential oil showed noteworthy antifungal activity against *Cryptococcus neoformans, Candida albicans*, and *Candida glabrata* and could be a potential candidate for further antifungal drug development.

## 1. Background

Fungi are eukaryotic organisms that cause a range of clinical infections including skin, hair, nail, mucosal, subcutaneous, and invasive infections. The majority of fungi cause opportunistic infections in immune-compromised people or individuals taking immune suppressive drugs [1, 2]. These include people with HIV/AIDS, primary immune deficiency, cancer chemotherapy, hematologic and solid organ transplantation, and prematurity and those on immune-modulatory medications [3]. More than 90% of reported fungal-associated deaths are caused by species belonging to three genera: *Cryptococcus*, *Candida*, and *Aspergillus* [4].

The burden of fungal infections is alarming in the world: more than 10 million people have mucosal candidiasis and more than 150 million people have serious fungal diseases, which have a major impact on their lives or are fatal [5]. Mortality associated with a fungal disease is more than 1.6 million, similar to that of tuberculosis and over 3-fold more than malaria [6]. The major concern of fungal infections is the high rate of mortality associated with invasive fungal infections, which often exceeds 50% despite the availability of antifungal drugs [4]. This is due to the emergence of widespread drug resistance against antifungal agents that limits treatment options [7].

Antifungal treatment options are highly limited, as there are few chemical classes represented by existing antifungal drugs [4]. To date, there are three different classes of antifungal agents, polyenes, azoles, and echinocandins [8]. Drug toxicity is the major limitation of polyenes and the older generation of azole antifungal agents. New antifungal agents (e.g., echinocandins and second-generation triazoles) in the past decade have transformed the management of invasive mycoses. These newer antifungal agents have also important limitations such as in pharmacokinetics, drug-drug interactions, and unusual toxicities associated with long-term use [9].

*Echinops kebericho* is an endemic medicinal plant widely used in Ethiopia. It is used to treat different infectious and noninfectious diseases such as fever, headache, stomachache, malaria, and cough in the community [10]. Extracts and essential oils of the roots of *E. kebericho* have been assessed for their antimicrobial [11], anthelminthic [12], and molluscicidal activities [13].

Essential oils are known for their antibacterial, antifungal, antiviral, insecticidal, and antioxidant properties [14]. They are widely used in traditional medicine and the food industry. The main constituents of essential oils are terpenes, terpenoids, aromatic and aliphatic constituents, and some hydrocarbons, which also exhibit antimicrobial effects [15]. Studies have shown that essential oils target the cell wall membrane, thereby disturbing ATP production and pH homeostasis [16]. A study conducted by Ameya et al. on the crude methanol and ethanol extracts of *E. kebericho* demonstrated activity against clinical isolates of *Candida albicans* and *Aspergillus flavus* [11]. Though the research was done on the crude extract against the two strains, the effect on the other fungal strains is unknown, and there has been no study on the essential oil of E. *kebericho*. The aim of this study, therefore, was to evaluate the antifungal activity of the essential oil of *E. kebericho* M. against *Candida albicans, Candida glabrata, Cryptococcus neoformans,* and *Aspergillus flavus*.

## 2. Methods

### 2.1. Study Design and Setting

This was an *in vitro* experimental study. The extraction of the plant essential oil was carried out at the Pharmacognosy Laboratory at Addis Ababa University. Determination of *in vitro* activity was done at the Mycology Unit of the Microbiology Laboratory at Mbarara University of Science and Technology. Clinical fungal strains of *C. albicans, C. glabrata, C. neoformans*, and *A. flavus* and quality control strains, *C. parapsilosis ATCC 22019 and C. krusei ATCC 6258,* were used for this study.

### 2.2. Chemicals and Equipment

For this study, we used 0.5 McFarland Standard, dimethyl sulphoxide (DMSO), Sabouraud's dextrose agar, RPMI 1640, potato dextrose agar, Mueller-Hinton agar, glucose, and methylene blue dye. The equipment used included a digital measuring balance, vortex, mortar, and pestle; and the apparatus included beakers, conical and round-bottom flasks, inoculating loops, pipettes, 96-well microtiter plates, densitometer, Clevenger apparatus, incubator, autoclave, and dry oven.

### 2.3. Plant Collection and Extraction

Fresh tubers of *E. kebericho* were collected from areas around Addis Ababa, Ethiopia. A taxonomist identified the plant and a voucher number of 001TB/19 was given and deposited at Addis Ababa University (AAU) Herbarium. The tubers were separated from the rest of the plant parts and washed. After air-drying in shade, the tuber material was pounded using a mortar and pestle to a fine powder; then 500 gm of the powder was dissolved in water in a 1 : 5 (weight-by-volume) ratio in a round-bottom flask and hydrodistilled for 3 hours using a Clevenger apparatus [17]. The volume of the essential oil obtained in the receiver was measured and stored in the refrigerator until used.

### 2.4. Determination of Antifungal Effect

#### 2.4.1. Preparation of Essential Oil for Antifungal Activity Assay

Essential oil (0.1 gram) was dissolved in 8 ml of 1% DMSO and filled up to a total solution of 10 ml with DMSO. A twofold dilution was prepared from this stock solution, which was filled in each well [18]. The EO of 10 mg/ml to 0.038 mg/ml concentration was used for the antifungal test.

#### 2.4.2. Well Diffusion Assay

Well diffusion assay for *Candida* spp. and *Cryptococcus* spp. was conducted according to M44-A Standard [19, 20]. Sabouraud's dextrose agar (SDA) media for yeast and potato dextrose agar were used for the subculturing of the A*. flavus* [20, 21]. Mueller-Hinton agar supplemented with 2% glucose and 0.5 *μ*g/ml methylene blue dye medium was used for testing of *Candida* spp. and *Cryptococcus* spp. [20]. Nonsupplemented Mueller-Hinton agar was used for testing of the *A. flavus* [21]. Fluconazole (0.25 mg/ml) was used as a positive control for *Candida* spp. and *Cryptococcus* spp. [22]. Amphotericin B (0.1 mg/ml) was used as a positive control for *A. flavus* [21]. For the negative control, 1% DMSO was used [11]. After the inoculation of the organisms, 8 mm wells were created and filled with 100 *μ*l of different concentrations of essential oil. Positive control (100 *μ*l) and negative control were also added [2]. All the plates were prepared in triplicate and incubated at 35°C for 24 hours for *Candida* spp. and 48 hours for *Cryptococcus* spp. At the end of the incubation period, the diameter of the zone of inhibition in each of the inoculated plates was measured and the diameter of the well was subtracted to determine the antifungal activities of the essential oil [2, 11, 18].

#### 2.4.3. Broth Microdilution

Broth microdilution was used to determine the minimum inhibitory concentration (MIC). Sabouraud's dextrose agar (SDA) media were used to subculture yeast following the recommendation of international guidelines [23]; for *A. flavus,* potato dextrose agar (PDA) media were used [21, 24]. The essential oil was prepared in serial dilutions in dimethyl sulphoxide (DMSO). 100 mg of *E. kebericho* essential oil was measured and DMSO was added to 1 ml total solution forming 100 mg/ml. Serial dilution was made in twofold dilution (1.56 mg/ml–100 mg/ml). Each serially diluted essential oil was further diluted in a ratio of 1 : 50 in RPMI media (100 *μ*l of stock solution was added to 4.9 ml of the RPMI), giving dilutions in the range of 0.03125 to 2 mg/ml. 100 *μ*l from each dilution was filled in the 96-well plates in accordance with CLSI protocol. The essential oil in the well was further diluted when the inoculum was added to the well to obtain dilutions in the range of 0.0156 mg/ml–1 mg/ml [23]. Fluconazole and amphotericin B were used as standard drugs for yeasts and Aspergillus, respectively. Twofold serial dilutions ranging from 0.25 to 128 *μ*g/ml for fluconazole and 0.031 to 16 *μ*g/ml for amphotericin B were used as a positive control; 1% DMSO was used as a negative control. Quality was controlled by using quality control strains: *C. parapsilosis* (ATCC 90018) and *C. krusei* (ATCC 6258) [25]. The MIC was the lowest concentration that inhibited the visible growth of the fungi.

#### 2.4.4. Inoculum Preparation

The inoculum was prepared according to CLSI guidelines [23, 24]: *C. albicans*, *C. glabrata*, *C. neoformans*, *C. parapsilosis* ATCC 22019, and *C. krusei* ATCC 6258 were subcultured on SDA media, while *A. flavus* was subcultured on PDA media. The inoculum was prepared from 24-hour-old colonies of *Candida* species. Approximately 1 mm diameter was suspended in 5 ml of saline and adjusted to 0.5 McFarland Standard. For *Cryptococcus,* the 72-hour-old colonies were suspended in 5 ml of saline and adjusted to 0.5 McFarland standards. The prepared suspension was vortexed for 15 minutes and the cell density was adjusted to 0.5 McFarland standards. This suspension was mixed for 15 seconds with a vortex, diluted to 1 : 50, and further diluted to 1 : 20 with RPMI medium to obtain the twofold test inoculum. The (twofold) inoculum was diluted when the wells were inoculated [23]. For *A. flavus,* the spores were harvested from 7-day-old cultures on PDA. The saline was poured into the PDA slant and mixed with a sterile swab. After 10 minutes of sedimentation, the suspension was transferred to a sterile test tube and adjusted to the 0.5 McFarland standards. This suspension was further diluted to 1 : 50 (100 *μ*l of 0.5 McFarland Standard was added to 4.9 ml RPMI media); 100 *μ*l of the diluted was then added to the well [24]. The minimal fungicidal concentrations (MFCs) were determined by subculturing fungal isolates from the wells with no visible growth onto SDA plates. The inoculated plates were incubated for 3 days at 35°C. MFC was the lowest concentration of drug that yielded a negative subculture [23, 26].

### 2.5. Statistical Analysis

The results were graphically presented as mean ± SD. Linear regression was used to determine the relationship between the concentration of the essential oil as an independent variable and zone of inhibition (response) as a dependent variable. Two-way analysis of variance was used to compare the effect of treatment using EO on the test microorganisms and to examine the difference in MIC of essential oil at different concentrations and fluconazole for each test strain. The independent variables were fungal strain and treatment (essential oil, fluconazole, and negative control). *p* value <0.05 was considered statistically significant. Statistical analysis was done using GraphPad Prism 5.01 software.

### 2.6. Ethical Considerations

The research protocol was approved by the Research Ethics Committee (REC) of Mbarara University of Science Technology (approval number 05/01-19).

## 3. Results

The percentage yield of the essential oil was 0.18% w/v of dried weight.

### 3.1. Agar Well Diffusion Assay

In agar well diffusion, wells filled with 100 *μ*l of different concentrations of EO produced inhibition zones ranging from 3 mm to 17 mm. The largest diameter was against *C. neoformans* and the lowest was against *C. krusei* ATCC 6258. The zone of inhibition of controls varied from 10 ± 2 mm to 22 ± 2 mm. The lowest diameter was produced by *A. flavus* and the widest was produced by *C. albicans* (Table 1). Two-way analysis of variance showed that both concentration and fungal species had significant effects (*p* < 0.0001) on the zone of inhibition (Table 1); for concentration, *F* = 39.08, df = 9, *p* < 0.0001; and for fungal species, *F* = 32.41, df = 4, *p* < 0.0001.

### 3.2. Minimum Inhibitory Concentration

**Figure 1:**
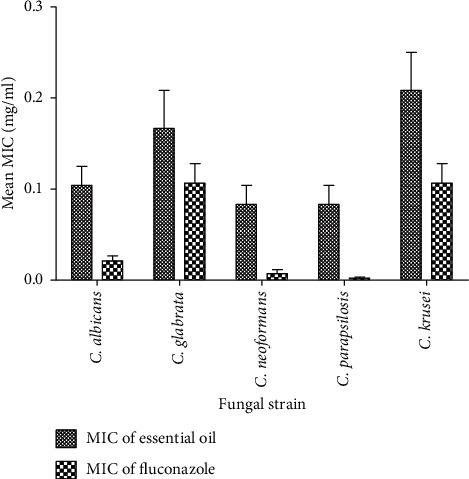
Comparison of the minimum inhibitory concentration of essential oil and fluconazole against fungal strains. Values are the means of the minimum inhibitory concentrations ± SD of three replicates (*N* = 3 replications).

The evaluation of the MIC of *E. kebericho* essential oil showed notable activity against all fungal strains tested except for *A. flavus*. *Candida parapsilosis* (ATCC 22019) and *Candida krusei* (ATCC 6258) which were used as quality controls had MICs in the recommended ranges. The MIC ranged from 0.083 mg/ml to 0.208 mg/ml. *Candida albicans* and *Cryptococcus neoformans* were highly sensitive, showing comparable activity to C. *parapsilosis*. *Aspergillus flavus* did not show sensitivity up to 1 mg/ml of the essential oil. There were no significant differences in MIC of essential oil and fluconazole against yeast (*p* value >0.05). The essential oil showed activity comparable to that of the standard drug (fluconazole) against *C. glabrata* and *C. krusei* (Figure 1).

**Figure 2:**
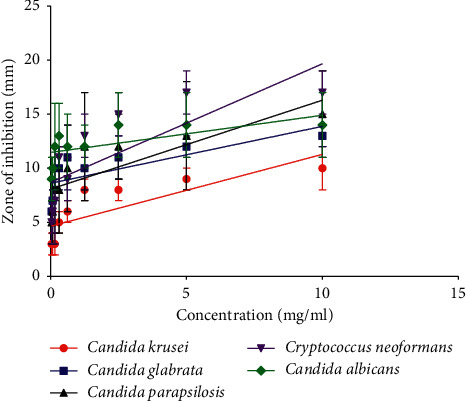
Linear regression showing the relationship between concentration and the zone of inhibition of essential oil for each strain. The straight line shows the line of best fit. The slope was significantly nonzero for all the strains, except *C. albicans* (*p* = 0.0091 for *C. krusei*; *p* = 0.0193 for *C. glabrata*; *p* = 0.0075 for *C. parapsilosis*; *p* = 0.0117 for *C. neoformans*; and *p* = 0.0626 for *C. albicans*). The *R*^2^ for *C. krusei* was 0.6452, for *C. glabrata* was 0.5660, for *C. parapsilosis* was 0.6633, for *C. neoformans* was 0.6210, and for *C. albicans* was 0.4115. Values shown are means ± SD; *n* = 3 replicates.

All the strains tested were susceptible to the standard drug (fluconazole for *Candida* species and *Cryptococcus neoformans*; amphotericin B for *A. flavus*). Linear regression showed that there was a positive relationship between concentration and zone of inhibition (Figure 2). The slopes for all, except *A. flavus*, were positive. The essential oil did not show a zone of inhibition against the isolate of *A. flavus*. The negative control (DMSO) did not show a zone of inhibition.

### 3.3. Minimum Fungicidal Concentration

**Figure 3:**
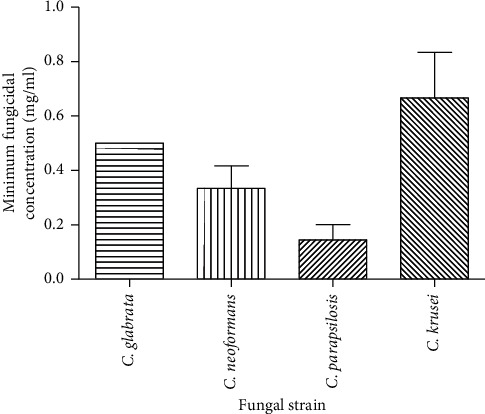
Minimum fungicidal concentrations of essential oil from *E. kebericho* against tested fungal strains. Values are the means of the minimum fungicidal concentrations ± SD of three replicates (*N* = 3 replications). The SD for *C. glabrata* was zero.

The essential oil showed the lowest MFC against *C*. *parapsilosis* and the highest MFC against *C*. *krusei* (Figure 3). The essential oil, however, did not show a fungicidal effect against *A. flavus* and *C. albicans* in the range of concentrations tested.

## 4. Discussion

The present study investigated the antifungal activity of the essential oil of *E. kebericho* on highly pathogenic invasive fungal strains, *C. albicans, C. glabrata, C. neoformans*, and *A. flavus* and quality control strains *C. parapsilosis* and *C. krusei*. The study demonstrated noteworthy antifungal activity of the essential oil of *E. kebericho* against the fungal strains: *C. neoformans* was highly sensitive to the essential oil of *E. kebericho*, followed by *C. albicans,* while *C. glabrata* was moderately sensitive. *Aspergillus flavus*, however, was not sensitive at all the tested concentrations. The zone of inhibition diameter decreased as the concentration of the essential oil decreased, showing a dose-dependent effect. The dose-dependent effect was similar to a previous study conducted with peppermint oil against fungal strains [27]. Fungicidal effects were observed against *C. glabrata, C. neoformans, C. krusei* ATCC 6258, and *C. parapsilosis* ATCC 22019.

Screening of medicinal plants for antifungal activity is important for novel drug discovery [28]. In many countries, fungal diseases have been treated using herbal remedies as many pathogenic fungi are becoming commonly resistant to numerous conventional antifungal drugs [29]. This study is the first of its kind to investigate the antifungal activity of essential oil from *E. kebericho,* although a previous study showed noteworthy antifungal activity using methanolic and ethanolic crude extracts of *E. kebericho* against *C. albicans* and *A. flavus* [11].

The activity of *E. kebericho* essential oil against the fungal strains studied varied with the fungal strain: better activity was observed against *C. neoformans* than the other strains. In a previous study, the essential oil of *Lavandula viridis* L. and Zingiberaceae species (*Z. officinale and Z. cassumunar*) showed noteworthy activity against *C*. *neoformans.* For *C. krusei* [30], a comparable result was also observed with the essential oil of *Cinnamomum zeylanicum* Blume [31]. Higher MIC was observed for *C. krusei* on control (fluconazole) and available antifungals [32]. A relatively higher MIC was also seen with *C. glabrata,* although it was less than that seen with *C. Krusei*. This was in agreement with a study conducted with the essential oil of *Artemisia sieberi* and *Origanum vulgare* [33]. *Candida krusei* is a multidrug-resistant fungal pathogen showing resistance with amphotericin B or fluconazole [34]. A study conducted on the essential oil of Thyme, *Summer savory*, and Clove against *A. flavus*, however, showed good antifungal activity against *A. flavus* [35]. This difference could be due to differences in the chemical constituents of essential oil between the plants, geographical variations, and/or genetic differences of the isolates used in the study [33].

The essential oil of *E. kebericho* possessed fungicidal activity against *C. glabrata, C. parapsilosis, C. krusei*, and *C. neoformans,* a finding also seen in a study conducted on *Thymus x viciosoi, Angelica major*, and *Artemisia sieberi* essential oil [33, 36, 37]. *E. kebericho* essential oil, however, showed MFC >1 mg/ml against *C. albicans*. In a recent study conducted on *Pistacia vera* L., the essential oil showed fungicidal effect against *C. albican*s at a concentration greater than 1 mg/ml, a finding which is in agreement with the present study [38]. In another study conducted by Devkatte et al. [39] on essential oils as potential inhibitors of *C. albicans* growth, out of 38 herbal oils, 9 oils (orange oil, rosemary oil, bergamot oil, clary sage oil, juniper oil, ginger oil, rose oil, Citronella oil, and Eucalyptus oil) also showed higher MFCs [39].

An analysis of the chemical composition of the essential oil of *E. kebericho* by Hymete et al. revealed sesquiterpenoid compounds as the main component [17]. These compounds could be responsible for the antifungal activity because of their highly lipophilic nature, their low molecular weights, and their capability of disrupting the cell membrane, causing cell death or inhibiting sporulation and germination [40, 41].

Two basic methods for the assessment of antimicrobial activities of essential oils are commonly used: the agar diffusion method (paper disc or well) and the broth dilution method. Although the use of microdilution in determining the activity of essential oil is the best way, agar diffusion could be utilized as a screening method, or in combination with a microdilution assay [2, 42]. In the present study, we used the microdilution method for the determination of MIC and MFC [2, 43]. Most of the antimicrobial molecules in plant extracts are significantly nonpolar and do not diffuse well in the aqueous agar matrix used in agar diffusion. The minimal inhibitory concentration (MIC) method remains the preferred one in the determination of the antimicrobial activity of plant extracts [44].

This study had a number of limitations. First, the study was *in vitro* and did not reflect the actual therapeutic effect that may arise from variations in pharmacokinetic and pharmacodynamic *in vivo* models. Second, the volatility of the essential oils could have reduced the activity. Third, the chemical composition of the essential was not studied though it has previously been studied. However, this study provides a broader insight for further *in vivo* studies that would also include specific experiments involving the isolated and identified active components of the essential oil of *E. kebericho*.

## 5. Conclusion and Recommendations

In the current study, the essential oil of *E. kebericho* showed noteworthy activity against *C. neoformans* and *C. albicans*. The activity was dose-dependent and fungicidal against *C. glabrata,* C*. neoforman*s, *C. parapsilosis*, and *C. krusei*. Further studies are warranted to evaluate the effectiveness of the essential oil *in vivo*, elaborate the mechanism of action, and isolate and identify the most active component(s). The activity of the combination of the essential oil with conventional antifungal drugs should also be investigated.

## Figures and Tables

**Table 1:** Antifungal activity of *Echinops kebericho* EO against pathogenic fungi using the agar well diffusion method. ND, Not determined; —, No zone of inhibition observed. ^*∗∗∗*^, *p* < 0.001 compared with *C. krusei*; ^*∗∗*^, *p* < 0.01 compared with *C. krusei*; ^*∗*^, *p* < 0.05 compared with *C. krusei*; ^###^, *p* < 0.001 compared with *C. krusei*; ^##^, *p* < 0.01 compared with *C. krusei*; ^#^, *p* < 0.05 compared with *C. krusei*. Values are the means of the zone of inhibition ± SD of three replicates (*N* = 3 replications).

Concentration (mg/ml)	Zones of inhibition (mm) (mean ± SD)					
	*Aspergillus flavus*	*Candida krusei*	*Candida glabrata*	*Candida parapsilosis*	*Cryptococcus neoformans*	*Candida albicans*
10	—	10 ± 2	13 ± 1	15 ± 4	17 ± 2^*∗∗*^	14 ± 3
5	—	9 ± 1	12 ± 1	13 ± 5	17 ± 2^*∗∗∗*^	14 ± 3
2.5	—	8 ± 1	11 ± 2	12 ± 3	15 ± 2^*∗∗*^	14 ± 3^#^
1.25	—	8 ± 1	10 ± 2	12 ± 5	13 ± 2	12 ± 2
0.625	—	6 ± 1	11 ± 3	10 ± 4	9 ± 2	12 ± 3^#^
0.313	—	5 ± 1	10 ± 2	8 ± 4	11 ± 1^*∗*^	13 ± 3^###^
0.156	—	3 ± 1	8 ± 1	8 ± 4	7 ± 4	12 ± 4^###^
0.078	—	3 ± 1	7 ± 1	6 ± 3	6 ± 2	10 ± 1^##^
0.039	—	3 ± 1	6 ± 0	5 ± 2	5 ± 2	9 ± 2^#^
Fluconazole	ND	12 ± 3	17 ± 3	22 ± 2	19 ± 3^*∗∗*^	22 ± 1^###^
Amphotericin B	15 ± 1	ND	ND	ND	ND	ND
DMSO	—	—	—	—	—	—

## Data Availability

The datasets used and/or analyzed during the current study are available from the corresponding author on request.

## Abbreviations

ATCC: American type culture collection
CLSI: Clinical laboratory standard institute
DMSO: Dimethyl sulphoxide
MFC: Minimum fungicidal concentration
MIC: Minimum inhibitory concentration
PDA: Potato dextrose agar
RPMI: Roswell park memorial institute medium
SDA: Sabouraud dextrose agar.

## Supplementary Materials

Supplementary Materials Supplementary Table: tabular results for linear regression analysis. Supplementary Figure: residual plot for the data.
