# Supplementary material for: Antifungal Activity of the Essential Oil of Echinops kebericho Mesfin: An In Vitro Study
Source: Evid Based Complement Alternat Med. 2020 Nov 12;2020:3101324. doi: 10.1155/2020/3101324 (PMC7676924; doi:10.1155/2020/3101324)
Supplement: Supplementary Materials — Supplementary Table: tabular results for linear regression analysis. Supplementary Figure: residual plot for the data. [file 3101324.f1.docx]

**Supplementary Table:** Tabular results for linear regression analysis

|  | C. krusei | C. glabrata | C. parapsilosis | C. neoformans | C. albicans |
| --- | --- | --- | --- | --- | --- |
| Best-fit values |  |  |  |  |  |
| Slope | 0.6641 ± 0.1862 | 0.5261 ± 0.1741 | 0.8227 ± 0.2216 | 1.098 ± 0.3242 | 0.3436 ± 0.1553 |
| Y-intercept when X=0.0 | 4.638 ± 0.7165 | 8.611 ± 0.6702 | 8.064 ± 0.8528 | 8.676 ± 1.248 | 11.46 ± 0.5978 |
| X-intercept when Y=0.0 | -6.984 | -16.37 | -9.802 | -7.902 | -33.35 |
| 1/slope | 1.506 | 1.901 | 1.215 | 0.9108 | 2.910 |
| 95% Confidence Intervals |  |  |  |  |  |
| Slope | 0.2239 to 1.104 | 0.1143 to 0.9379 | 0.2987 to 1.347 | 0.3312 to 1.865 | -0.02369 to 0.7110 |
| Y-intercept when X=0.0 | 2.944 to 6.333 | 7.026 to 10.20 | 6.047 to 10.08 | 5.725 to 11.63 | 10.05 to 12.87 |
| X-intercept when Y=0.0 | -25.43 to -2.965 | -83.50 to -7.999 | -31.15 to -4.865 | -31.67 to -3.404 | -infinity to -14.82 |
| Goodness of Fit |  |  |  |  |  |
| r² | 0.6452 | 0.5660 | 0.6633 | 0.6210 | 0.4115 |
| Sy.x | 1.757 | 1.643 | 2.091 | 3.060 | 1.466 |
| Is slope significantly non-zero? |  |  |  |  |  |
| F | 12.73 | 9.131 | 13.79 | 11.47 | 4.895 |
| DFn, DFd | 1.000, 7.000 | 1.000, 7.000 | 1.000, 7.000 | 1.000, 7.000 | 1.000, 7.000 |
| P value | 0.0091 | 0.0193 | 0.0075 | 0.0117 | 0.0626 |
| Deviation from zero? | Significant | Significant | Significant | Significant | Not Significant |
| Data |  |  |  |  |  |
| Number of X values | 9 | 9 | 9 | 9 | 9 |
| Maximum number of Y replicates | 1 | 1 | 1 | 1 | 1 |
| Total number of values | 9 | 9 | 9 | 9 | 9 |
| Number of missing values | 0 | 0 | 0 | 0 | 0 |
| Runs test |  |  |  |  |  |
| Points above line | 5 | 5 | 4 | 4 | 6 |
| Points below line | 4 | 4 | 5 | 5 | 3 |
| Number of runs | 3 | 3 | 3 | 5 | 3 |
| P value (runs test) | 0.0714 | 0.0714 | 0.0714 | 0.5000 | 0.1071 |
| Deviation from linearity | Not Significant | Not Significant | Not Significant | Not Significant | Not Significant |

**Supplementary figure:** Residual plot for the data
